# The implementation of change model adds value to value-based healthcare: a qualitative study

**DOI:** 10.1186/s12913-019-4498-y

**Published:** 2019-09-06

**Authors:** Nina Zipfel, Paul B. van der Nat, Benno J. W. M. Rensing, Edgar J. Daeter, Gert P. Westert, A. Stef Groenewoud

**Affiliations:** 1 Department of Value-based Healthcare, St. Antonius Hospital, P.O. Box 2500, 3430 EM Nieuwegein, the Netherlands; 2 Radboud university medical center, Radboud Institute for Health Sciences, Scientific Center for Quality of Healthcare (IQ healthcare), P.O. Box 9101, 6500 HB Nijmegen, the Netherlands; 3 Department of Cardiology, St. Antonius Hospital, P.O. Box 2500, 3430 EM Nieuwegein, the Netherlands; 4 Department of Cardiothoracic Surgery, St. Antonius Hospital, P.O. Box 2500, 3430 EM Nieuwegein, the Netherlands

**Keywords:** Implementation science, Implementation of change model, Value-based healthcare, Outcome improvement, Outcome measurement

## Abstract

**Background:**

Value-based healthcare (VBHC) is a concept that focuses on outcome measurement to contribute to quality improvement. However, VBHC does not offer a systematic approach for implementing improvement as implementation science does. The aim is to, firstly, investigate the implementation of improvement initiatives in the context of VBHC and secondly, to explore how implementation science could be of added value for VBHC and vice versa.

**Methods:**

A case study with two cases in heart care was conducted; one without the explicit use of a systematic implementation method and the other one with the use of the Implementation of Change Model (ICM). Triangulation of data from document research, semi-structured interviews and a focus group was applied to evaluate the degree of method uptake. Interviews were held with experts involved in the implementation of Case 1 (*N* = 4) and Case 2 (*N* = 7). The focus group was held with experts also involved in the interviews (N = 4). A theory-driven qualitative analysis was conducted using the ICM as a framework.

**Results:**

In both cases, outcome measures were seen as an important starting point for the implementation and for monitoring change. Several themes were identified as most important: support, personal importance, involvement, leadership, climate and continuous monitoring. Success factors included intrinsic motivation for the change, speed of implementation, complexity and continuous evaluation.

**Conclusion:**

Application of the ICM facilitates successful implementation of quality- improvement initiatives within VBHC. However, the practical use of the ICM shows an emphasis on processes. We recommend that monitoring of outcomes be added as an essential part of the ICM. In the discussion, we propose an implementation model that integrates ICM within VBHC.

**Electronic supplementary material:**

The online version of this article (10.1186/s12913-019-4498-y) contains supplementary material, which is available to authorized users.

## Contributions to the literature

- Value-based healthcare (VBHC) focuses on outcome measurement to contribute to quality improvement. VBHC does not offer an implementation methodology for improvement initiatives as the Implementation of Change Model (ICM) does.
- Outcome measurement is an important starting point.
- Support, personal importance, involvement, leadership, climate and continuous monitoring are important for implementation.
- Success factors included intrinsic motivation for the change, speed of implementation, complexity and continuous evaluation.
- An Integrated Implementation Model is proposed for the implementation of VBHC improvement initiatives that incorporates monitoring of outcome measures and integrates the ICM.

## Background

Improving the quality of care while reducing costs is a major goal on many hospital agendas [1, 2]. The goal of value-based healthcare (VBHC) is to reorganize health care in order to increase value for patients [3]. ‘Value’ in VBHC is defined as patient-relevant health outcomes relative to costs [3]. Porter suggests that this goal can be achieved by measuring outcomes and costs per medical condition, which will allow for the identification of variation in outcomes across the full cycle of care [4]. Experts suggest that, based on this insight into outcomes, improvement potential can be identified and quality of care improved [5]. In current practice, VBHC is used as a concept leading to improvement by measuring outcomes in registries and supporting more efficient coordination of care through benchmarking and reporting [6]. However, the current application of VBHC lacks a systematic approach for the implementation of improvements. The concept is sometimes presented as the sole solution for improving outcomes and reducing costs, but *how* improvements should be implemented remains unclear. In the literature, a lack of a systematic approach for using VBHC and specifically a method for the implementation of improvement initiatives was identified [1]. Measurement of outcomes and costs has been shown to provide valuable insights into practice variation and waste, which can lead to process improvement [7, 8]. Literature on the implementation of improvement initiatives in the context of VBHC is scarce. One example was identified in the context of a project for orthopaedics, in which the identification of variation in hospital stay led to improvement [7]. Another example, which involved prostate cancer care, showed that improvement based on outcomes led to a relevant decrease in incontinence rates [9]. Moreover, within heart care several improvement initiatives were implemented based on identified variation in outcomes [1]. How the improvements were implemented was, however, not described. Therefore, we aimed to investigate the implementation of improvement initiatives in the context of VBHC and whether a systematic implementation method has added-value for VBHC. The resulting insight could enrich the concept of VBHC [10].

In order to investigate whether systematic implementation could add value to VBHC, a suitable framework needed to be identified. A previous review identified implementation frameworks, models and theories for the process of implementation [11]. The most commonly cited frameworks include the PARIHS, [12] Conceptual Model, [13] the Implementation of Change Model, [14] Ecological Framework [15] and the CFIR. [16] Based on the results of this review, the Implementation of Change Model (ICM) seemed to be the most suitable for the purpose of offering a systematic approach for the implementation of improvements since it specifies practical steps for the process of implementation [14]. Several quality improvement projects have applied the ICM or parts of it [17–19].

This paper describes how improvement initiatives which were selected based on insights into outcomes were implemented. To show the added-value of a systematic implementation approach for VBHC, we selected two cases. The goal was to use VBHC as a guideline for both projects in the identification and selection of an improvement intervention. Both interventions emerged from a VBHC improvement cycle. In an earlier systematic literature review only very few improvement interventions based on insights into outcomes were identified [20]. Therefore, the aim was to compare two improvement interventions that used the same starting point to compare the implementation process. The first case was implemented *without* the explicit use of a systematic implementation approach, while the second case was implemented *with* the explicit use of a systematic implementation approach, i.e. the ICM. By analysing and comparing the two cases, the goal of this paper was to learn what went well and what could be improved in order to give recommendations on how to implement improvement initiatives in the context of VBHC. The analysis was not intended to evaluate the improvement on outcomes, but to explore the implementation process of two improvement initiatives.

### Theoretical framework

#### ICM

The ICM was developed based on examples from the practice of implementing change in health care and examples from the literature [21]. The ICM consists of seven steps for guiding the implementation of improvement (Table 1). The first step of the model is development of a proposal and target for change, which includes a detailed analysis of the characteristics of the possible innovation and/or change. Secondly, actual performance or outcome variation at baseline has to be assessed in order to gain insights into the current situation and indications for change [21]. The following step of the ICM is the problem analysis, which is seen as a crucial step to the implementation of an improvement initiative [14]. The analysis of barriers and facilitators should include a structured analysis of relevant stakeholders, determinants of change, and subgroups in the target population [22]. Based on the analysis of possible barriers, implementation strategies can be identified [21]. This step is followed by a pilot implementation and the integration into routine care [21]. The last step of the model is the evaluation of the change, which could lead to modifications and a return to earlier steps of the model [21]

**Table 1:** Seven steps of the ICM

Step	Principles of the ICM^1^
1.	Development of a proposal for change
2.	Analysis of actual performance, targets for change
3.	Problem analysis of target group and setting
4.	Development and selection of strategies and measures to change practice
5.	Development, testing and execution of implementation plan
6.	Integration of changes into routine care
7.	(Continuous) evaluation and (where necessary) adapting plan

1. Adapted from Grol et al. (2013). [14]

.

## Methods

### Design: case study

A collective case study design was chosen to test the ICM for two cases. Cases in collective case studies are similar, yet can have a different context [23]. The goal of a collective case study is to compare two or more cases [24]. For this analysis, a within-site collective case study was conducted. According to Creswell (1998), theory can be employed in different ways in a case study design: before or after data collection [23]. For the purposes of exploring the application of the ICM, theory was employed both for supporting the interview guide and for comparing both cases for interpretation after the interview. For this study the Consolidated criteria for reporting qualitative studies (COREQ) were applied.

### Case selection and setting

The two cases were selected according to the principles of purposive sampling [23]. Purposive sampling can be used to identify cases that show different perspectives on the same problem [23]. To provide a clear comparison of the implementation approaches used in the two cases, deviant case sampling was applied. In deviant case sampling, cases are selected that are contrasting in some way [25]. For our purposes, one case was chosen that implemented an improvement initiative without the explicit application of a systematic implementation method, while in the other case there was explicit use of the ICM. Both cases emerged based on insights into outcomes according to the VBHC concept. The starting point for the development of both improvement initiatives was the same set of outcome measures and both initiatives share the goal of improving these outcomes. Therefore, both cases are comparable due to their context, yet they can also be contrasted. Creswell (1998) suggests that the more cases are studied, the less depth the cases have [23]. Only two cases were chosen to ensure that they were “information rich”; it was not the purpose of the study to achieve statistical generalization [26]. The research was carried out at a Dutch hospital from June 2017 until January 2018. The first selected case concerns a pre-incision checklist for cardiac surgery to improve cultural behaviour in the operating theatre and reduce 120-day mortality rate (Table 2). The second case is about a protein-enriched diet given to patients two weeks before the operation in order to improve their fitness before cardiac surgery and prevent postoperative complications (Table 2).

**Table 2:** Description of the cases

Case 1: A pre-incision checklist for cardiac surgery	Case 2: Preoperative protein-enriched diet
The pre-incision checklist for cardiac surgery is an addition to the surgical safety checklist that was previously developed by the World Health Organization [27]. Items specific to cardiac surgery are added to the checklist and patients are divided into three risk categories: low, intermediate and high risk. Peri- or postoperative complications are identified with a focus on six main organ-specific topics: cardiac, pulmonary, renal, neurologic, inflammation and coagulation. The checklist is part of a greater project from an external hospital that identified this “best practice” based on insights into outcomes [28]. The checklist was identified based on differences in 120-day mortality rate among benchmarking hospitals. [1] This external project is expected to contribute positively to communication between various members of the operation team. This is expected to contribute to more risk awareness, structured consultation and a better culture [28]. Evidence has shown that the checklist contributes to significantly lower 120-day mortality rate compared to a group of patients who did not receive the checklist [29]. At the current research setting only questions from the pre-incision checklist were implemented. The goal of the intervention was to improve outcomes (120-day mortality rate).	Elderly patients undergoing Surgical Aortic Valve Replacement (SAVR) or Transcatheter Aortic Valve Replacement (TAVR) receive a protein-enriched diet during a two-week period prior to the scheduled surgery. Offering a preoperative protein-enriched diet had a positive effect on health outcomes in cancer patients, patients with hip fracture undergoing surgery and patients with end-stage liver disease who needed to undergo transplantation [30–33]. In a study of non-cancer patients, malnutrition was most frequently identified in patients undergoing major vascular surgery [34]. The initiative was selected based on insights into outcomes and in-depth data and process analyses with the goal of optimizing preoperative preparation of older patients. The diet consists of familiar foods enriched with protein in order to reach the recommended protein intake for elderly people with an illness of 1.2–1.5 g/kg/d during and after hospitalization [35]. The goal is to increase protein intake by 45 g per day spread over meals during the day. Protein intake is measured with validated 24-h recall questionnaires. The protein-enriched diet is expected to contribute to higher protein intake, fewer postoperative complications and faster recovery. The effect of a preoperative protein-enriched diet for elderly patients undergoing aortic valve replacement is currently being evaluated.

### Data collection methods

Triangulation of data sources was applied. Using multiple sources for data collection is advised for case studies [23]. First, a document analysis of minutes, presentations and memos was conducted. The documents were made available by a member involved during each of the implementation processes per case. Second, interviews were conducted with professionals involved in the implementation process of the two selected cases. Interviews were semi-structured with a length of approximately 20 min. An interview guide based on the theoretical framework including the ICM was used (see Additional file 1). All interviews were audio-recorded and transcribed verbatim. Third, a focus group interview was conducted in order to recapitulate the results from the interviews. The focus group was intended for feedback purposes and gathering perceptions, and it allowed participants to make additional comments on each other’s opinions [25]. The focus group was audio-recorded and transcribed. The interviews and focus group were conducted by a researcher. The language of the interviews and focus group was Dutch and transcripts were translated into English.

### Sampling of participants

Participants for the interviews were selected through a mix of criterion sampling and snowball sampling. Criterion sampling is a method of choosing all participants that meet a predefined criterion [25]. The criterion for selection was that participants must have had an active role in the implementation of the case. Additional snowball sampling [25] was applied by asking participants whether other participants were involved in the implementation process who could provide more information. Participants were asked to participate via e-mail. For the first case, four professionals were chosen (*N* = 4) including a cardio-thoracic surgeon, a perfusionist, an anaesthesiologist and a data manager. This was the maximum number fulfilling the criterion, including participants suggested through snowball sampling, because no other participants were involved in the implementation of the intervention. For the second case, seven interviews were conducted (*N* = 7) with two cardiologists, a cardio-thoracic surgeon, a nurse, a researcher and two secretaries. Also for this case the maximum number of participants was chosen through both sampling strategies. The sample size was chosen because it was not the purpose of the cases to draw externally generalizable conclusions, but instead to collect all possible viewpoints, opinions and thoughts of relevant stakeholders about the case [25]. Informed consent was obtained from all participants before the start of the interviews.

The same participants from both cases were also invited to participate in the focus group in order to comprise a multidisciplinary group of experts from both cases. Convenience sampling was applied. This setup was chosen because participants could relate to comments made by colleagues since they shared experiences during the implementation process [36]. Four experts agreed to participate in the focus group (*N* = 4). Two experts were involved in the implementation of Case 1 and two of Case 2.

### Data analysis

We analysed the results in three steps: 1) Chronological case description with a within-case analysis from the documents and interviews, 2) Cross-case analysis from the interviews, and 3) Focus group analysis.

As recommended by Creswell (1998), the detailed case description is done chronologically [23]. The advantage of this approach is that each case can be described separately in order to understand each case as a holistic entity [25]. For this analysis both the documents as well as the interviews were used. The interviews were coded by one researcher. Subsequently, a within-case analysis was conducted with a detailed description of each case and themes within the case [23]. Each case can be seen separately as holistic and context sensitive [25]. A holistic perspective, according to Patton (2002), is one in which the whole context is seen as a complex system [25]. Thus, only when all interviews and sources from the document analysis are combined the whole case is formed. Context sensitivity refers to comparative case analysis and identifying patterns for transferability to a different setting [25]. Data for this analysis were gathered through document analysis and interviews.

A thematic analysis across cases was then carried out, which is known as a cross-case analysis [23]. The data were analysed using deductive analysis techniques based on the theoretical framework of the ICM [25]. In order to contrast and compare the cases, constant comparative analysis was applied [25]. Qualitative comparative analysis seeks to compare cases in order to generate explanations. For the analysis, a so-called truth table was developed in order to test the absence or presence of each step of the ICM [25]. The goal of this analysis approach was not to force the data into predetermined categories, but to show that the ICM enhances the knowledge of the implementation process of both cases. For this analysis the interviews were used. Subsequently, the focus group was analysed by comparing discussions of similar themes [36]. Both cases were interpreted in terms of success factors. Successful implementation was defined as a positive experience by participants.

Analyses were performed in atlas.ti 7.0.

The results of the case analysis are presented in three steps: 1) a chronological case description with a within-case analysis, 2) a cross-case analysis, and 3) the focus group analysis resulting in success factors for the implementation.

## Results

The interviews and focus group were held by a researcher (the primary researcher of the study).

### Case descriptions

A reconstruction of both cases was made.

**Fig. 1:**
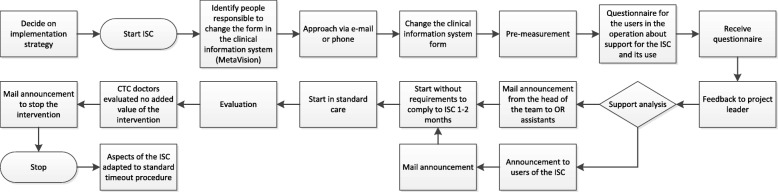
Flowchart of the implementation process of Case 1. Describes the process of the implementation of Case 1

Chronological steps of Case 1 with a within-case analysis (introduction of a pre-incision checklist for cardiac surgery in a hospital) (Fig. 1):

- The *proposal for change* was derived from results of another partnering hospital. At the partnering hospital a larger project was initiated, which included a pre-incision checklist. That hospital presented favourable outcomes in a benchmarking analysis with other Dutch heart centres, which was underpinned by the results of the document analysis. Insights into explanations for the differences in patient-relevant outcome measures showed that a pre-incision checklist could contribute to a reduction of 120-day mortality rate.
- The initial start of Case 1 took place with a pilot phase without the requirement to comply with the intervention. No clear *implementation team* was in place to inform potential participants about the use of the intervention, which left users unaware of its existence and added-value.
- It was reported that an intervention was started, but it was not carried out as a standard part of the care process. An analysis through questionnaires was carried out in the beginning to investigate whether the initiative was considered important and whether the *culture and context* would be open to it. The questionnaire included questions on the importance of the checklist to the users and the climate for implementing the checklist which resulted from the document analysis. Questions focused on how long employees have been working at the hospital, whether they thought that colleagues in the ward were treated with respect, whether they felt that they can tell when something is not going well in the operation room, whether they agreed with the introduction of the pre-incision checklist and whether the timing of the check just before incision of the patient was right. These questions were comparable to the validated Team Climate Inventory (TCI), which is an instrument used to measure organizational climate and team building and development [37].
- It was reported that the implementation took place very fast and time was needed to carry out more analyses and develop appropriate implementation strategies. A brief *implementation plan* was offered from an external partnership that had previously developed and implemented the initiative.
- Next, the checklist was announced during a *team meeting* of cardio-thoracic surgeons and disseminated via e-mail to operation assistants. The dissemination was supposed to happen from within. Thus, doctors and an anaesthesiologist were the primary contact persons in order to facilitate a low-threshold for asking questions and to prevent resistance from the people applying the checklist.
- A period of *voluntary participation* with regard to applying the checklist for cardiac surgery was established for about one to two months.
- In the subsequent step, the checklist was implemented in *routine care*. The implementation took place with a simple start and communication among involved colleagues.
- An *evaluation* followed, which led to the conclusion of cardio-thoracic surgeons in the hospital that the initiative did not add value to their work. An e-mail was sent to all involved parties and the checklist was stopped. However, some questions that are part of the checklist were integrated into the standard time-out form of the hospital.

The implementation process of Case 1 took place between December 2015 and February 2016.

**Fig. 2:**
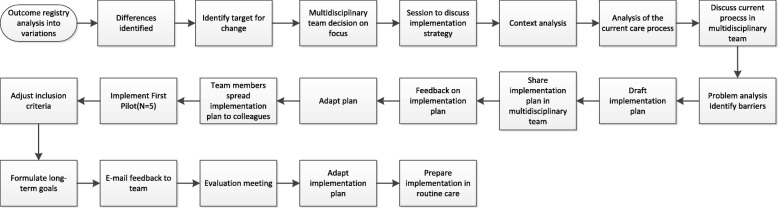
Flowchart of the implementation process of Case 2. Describes the process of the implementation of Case 2

Chronological steps of Case 2 with a within-case analysis (preoperative protein-enriched diet) (Fig. 2):

- The implementation process started with an *outcome analysis as a basis* for the target. The analysis was based on an outcome registry. Results of the analysis of outcome measures of the hospital made participants feel an urge to change with the development of an improvement intervention. The analysis resulted in a clear target. The target was considered feasible, but difficult to combine with the aims and wishes of the patients to receive an operation as fast as possible.
- The protein-enriched diet and the number of patients with undernutrition were analyzed and discussed in a multidisciplinary *implementation team*. The team consisted of a researcher, cardiologist, cardio-thoracic surgeon, anaesthesiologist, dietician, head of the hospital kitchen, nurse and researcher.
- The target was refined and a *context analysis* conducted. The context analysis included an analysis of the current preoperative process for older patients. This analysis could lead to a delay in implementation. The context analysis was conducted and discussed in the implementation team, but not further disseminated to all participants involved with the initiative in order to increase support for the implementation. The context analysis was done in several steps to gain as much insight as possible into the current process of care. This analysis formed the basis for the *problem analysis* to identify possible barriers and develop an implementation plan.
- The *implementation plan* was drafted. The implementation plan included a financial plan. It also led to the development of implementation strategies. The implementation plan was adjusted based on feedback from the implementation team. After adjustments, each team member disseminated the implementation plan to the broader involved team in the hospital. Individuals were offered training on their future tasks concerning the implementation.
- Subsequently, the protein-enriched diet was implemented in the form of a *pilot* aimed at including five patients.
- After these inclusions, an *evaluation meeting* was organized with the implementation team. The implementation plan and inclusion criteria were adjusted and long-term goals were formulated.
- In the subsequent step, *continuous feedback* was given via e-mail followed by another evaluation meeting with the implementation team where first results and outcomes were monitored. Following this meeting, adjustments to the plan were made and *implementation in routine care* was prepared.

The implementation of Case 2 took place between April 2016 and February 2017.

### Cross-case analysis

Each case was tested against the ICM. Table 3 summarizes to what extent both cases were implemented according to the steps of the ICM.

**Table 3:** Checklist whether the steps of ICM have been applied per case

		Case 1	Case 2
ICM	Development of a proposal for change	Χ	✓
	Analysis of actual performance	✓	✓
	Problem analysis of target group and setting	✓	✓
	Development and selection of strategies and measures to change practice	✓	✓
	Development, testing and execution of the implementation plan	Χ	✓
	Integration of changes into routine care	✓	X
	Continuous evaluation and adapting plan	Χ	✓

The first case was implemented without the application of a specific implementation approach, unlike the second case which used the ICM. Differences in the processes of both cases, as described in the case descriptions, included the development of a proposal for change, elaboration of an implementation plan, development of implementation strategies, testing and execution of the implementation plan and implementation into routine care. For the first case, the proposal for change was imposed by an external hospital through a network of hospitals [1]. Whereas, for the second case a detailed outcome analysis was conducted together with health care professionals who proposed to implement change based on the results of the analysis. The implementation of Case 1 started directly with a pilot phase followed by a culture and context analysis. An implementation plan was used from the external hospital, which was transferred to the current setting without adjustments. In Case 2, after formation of an implementation team, a detailed context analysis was conducted followed by drafting of an implementation plan suitable to the context. Concerning the implementation strategies, in Case 1 an announcement of the intervention during a team meeting and dissemination via e-mail was considered sufficient. In Case 2, the implementation plan was offered to individuals that would be affected by the intervention. The individuals were given the chance to comment and receive training on their tasks for the execution of the intervention. Furthermore, Case 1 was implemented into routine care after a short period of voluntary participation. The second case was not, yet, implemented into routine care, since evidence on the effect on outcomes was desired for the intervention to be implemented into routine care. In Case 1, no further interim evaluations took place. Only an end evaluation determining the stop of the intervention was organized. In comparison, for Case 2 continuous feedback was given followed by an evaluation meeting.

In the case comparison, a number of themes have been identified as most important for the implementation of improvement interventions with a focus on monitoring value (Table 4). These themes showed that the steps of the ICM enhance the implementation process of both cases.

- Support: Support is important in the beginning of the implementation and includes support for the proposal for change, but also for execution of the implementation plan. Support can also be linked to other steps of the ICM later on in the process, such as involvement and leadership.
- Personal importance of the target: Respondents mentioned that when an initiative feels important to them, the implementation process is improved.
- Involvement: For the problem analysis, involvement has been identified as a theme. For the first case, involvement was lacking and neither outcomes nor progress were shared with all participants involved in the initiative. That led to frustration and less uptake of the initiative.
- Leadership: Participants mentioned that there was no clarity on how to use the intervention in Case 1. This should have been resolved by having one leader in the operation room. That leader was not clearly defined and did not clearly perform his tasks. Therefore, the initiative lacked uptake.
- Climate: Development and maintenance of a positive climate were mentioned as being important for successful implementation. Room for critique and adjustment should be present.
- Monitoring: Monitoring, as part of the last step of the ICM, has been identified as important. Monitoring in the first case would have supported uptake as well. As mentioned by R2, if it had been monitored how often the checklist was used, it would have been possible to intervene faster.

**Table 4:** Results of the cross-case analysis

Steps of the ICM	Theme that emerged from cross-case analysis	Representative quotations
1. Development of a proposal for change	Support	R6:“The people who perform it are often not involved in such a thing.”
		R2: “So you are asking for extra commitment from people; if you ask, you also have to return something. If that does not happen, and there is not much support in advance, then it will break down.”
2. Analysis of actual performance	Personal importance of the target	R7:“We have all looked at whether this is a feasible goal and how can we do it all based on the analysis we had.”
		R5: “Certainly, the goal is that every patient who is undergoing an aortic valve replacement receives a protein-enriched diet (…). That it becomes a standard of care is actually the goal; it must be a standard concern.”
3. Problem analysis of target group and setting	Involvement	R2: “So that you’re involved, that you should receive the result, so that’s important”
		R6: “I was always kept up to date, so that was nice”.
		R2: “Yes, I think it’s important that everyone is involved. In particular, because if it does not happen, or someone forgets or does not feel like it, or quickly wants to do it, that someone in that operation room, even if it’s the operation nurse, can say: ‘Hey, those questions should also be asked.’ If the whole team knows that the question has to come up, they will do it, but if only the surgeon knows and he forgets, you think: yes, it happened again.”
		R2: “I think in advance, everyone’s role should be clearer, not just the one who does it, the surgeon and the anaesthesiologist, but also the others.”
4. Development and selection of strategies and measures to change practice	Leadership	R2: “So in the group, that is certainly decisive in the operation room, there was a difference in opinion that did not really help. If all surgeons would say: ‘No, we should definitely do that’, that is important.”
5. Development, testing and execution of the implementation plan	Climate	R1: “I think that the climate is good and that people feel free to indicate that. That is also one of the prerequisites for successfully implementing something like this, that every player on the team is free and feels free to simply say what he or she thinks.”
6. Integration of changes into routine care	No theme across cases identified	Not applicable
7. Continuous evaluation and adapting plan	Monitoring	R2: “If you see after two weeks that only half of the patients have been done, you should say: It was only 50%; it should improve. And then you have to go back two weeks later to make sure that you get 60%. Otherwise, you have to talk to people about: How did this happen?”
		R3: “We have been sitting extensively on those Thursdays, what should change to improve the success of the implementation and whether there are additional patient groups that can be included.”
		R8: “Yes, sometimes sending a mail like: guys, remember it.”

These themes are linked to steps of the ICM in order to see whether the ICM has added-value for the implementation of improvement initiatives in the context of VBHC. However, for one step of the ICM, namely step 6. Integration of changes into routine, no important theme across cases was identified.

### Focus group analysis: success factors for the implementation of improvement interventions

A focus group interview was conducted with four professionals who were also involved in the earlier interviews to critically reflect on the results of the interviews. Several success factors were identified: intrinsic versus extrinsic motivation, a multi-centre intervention compared to a single-centre intervention, the name of the intervention, speed of the implementation process, complexity, continuous feedback and output.

Firstly, the aspect participants reflected on was the fact that the motivation for successful implementation differs when the *motivation is extrinsic*, i.e. an intervention that is adapted from another hospital versus an intervention that the hospital developed itself. Adapting an external improvement intervention could potentially lead to social pressure for implementation, which could impact success.

“That of course makes a difference whether you invented something yourself and have time to roll it out or if you adapt something from outside. If you really want to participate, then you have to start before a certain date. Otherwise we are too late. That is missing here.” (R4)

Secondly, the *name of the intervention* which includes the name of another hospital has an impact on the success of the implementation, as elaborated by R7:

“Yes, or what you call it. I hear you call it differently. What is the difference from the original name? So then it is just what you call the intervention, because maybe you do it the right way, but you just call it a number of things under a different name. If it would have been called a different name, maybe we would have been more willing to apply it.”

Thirdly, the *speed* of an implementation is dependent on whether the intervention involves a multidisciplinary team or a smaller team. Participants mentioned that a systematic implementation model would be applicable for straightforward interventions, but decisions have to be made for interventions that require more extensive research in order to follow.

“The first case is something you have to implement with a whole team; it’s multidisciplinary. You have to get the anaesthesiologist, all participants of the time-out, the perfusion, the nurse, everyone has to support it. So many people need to say yes; I don’t see this happening.” (R4)

Fourthly, the *complexity* of an intervention influences the success and speed of the implementation. An intervention that is less complex would not need to follow all the steps of the model.

“I think if you do something with some kind of work agreement – so this is a work agreement that, for example, you only let members of the medical staff operate – then you need to follow fewer of those steps. I mean, it’s something you do that you agreed on with the whole team. But if you do something like Case 2 where you also have to measure things, then you have to start with the measurement. You have to organize something for recording the outcome (…) and have good data, and then yes, develop a proposal for an improvement. Yes, that sometimes starts before step 1.” (R4)

Fifthly, *continuous evaluation* of outcome measures can be time-consuming, but are also crucial for successful implementation and support. Participants also discussed continuous feedback.

“But shouldn’t you let the proposal for change come back continuously because that is at step 3, then data analysis, problem analysis. If you are going to implement strategies, then you actually want to see what effect it has. (…) Because if you have implemented your number of things, then you actually want to know what is the effect of that. And maybe it has no effect. So I would repeat the proposal for change more frequently.” (R4)

The participants noted that it could also be necessary to return from one step to the beginning in a systematic implementation model.

Sixthly, *output* was also mentioned as being important for successful monitoring. Output, the goal of a successful implementation, should be defined before implementation and, next to outcome measures, be evaluated continuously.

“This is not even outcome, it is output. In terms of input, throughput, output, outcome. I always make the comparison with a vaccination program. Output is how many people you vaccinate, and the outcome is the observed decrease in the prevalence of a condition in an area.” (R7)

The focus group interview identified six success factors for the implementation of improvement initiatives in the context of VBHC.

## Discussion

The study had three objectives: to investigate the implementation of improvement initiatives in the context of VBHC, to explore how implementation science could be of added-value for VBHC and vice versa, and to investigate what we can learn from the implementation of two cases in the context of VBHC. To accomplish these objectives, we compared two cases, one that used the ICM and one that did not. In this study, we showed that the use of an implementation model such as the ICM contributed to a more positive experience of the implementation team and better uptake.

Our study identified important themes for the implementation of improvement initiatives in the cross-case analysis. The factors identified in this study are in line with previous research from implementation science. Grol et al. also identified incentives for uptake in relation to the ICM steps, which include conveying a positive attitude towards change and motivation [38]. The literature identified the practitioners’ or users’ support the change as a leading factor for guideline adherence [39]. In our study, personal and professional involvement in the design of the intervention played an important role in the success of the implementation. Previous research also identified involvement as a success factor for uptake of clinical practice guidelines [39, 40]. Implementation of guidelines is comparable to implementation of improvement interventions. Furthermore, participants mentioned the importance of strong leadership and a climate in which users feel free to discuss issues. Incentives for change can be established at various levels, such as in the social context [38]. In the literature, the social context includes culture, leadership and collaboration [38]. The absence of social norms can hinder uptake [41]. Moreover, the composition of an improvement team should be diverse and include all relevant healthcare professionals, as noted in a systematic review of factors influencing guideline implementation [42].

The implementations themselves were considered successful based on the results of the interviews. The implementation process was experienced more positively for Case 2, even though it was not yet implemented into routine care. Nevertheless, the implementation of Case 2 does not yet show an effect on relevant outcome measures. The themes that emerged from the cross-case analysis indicate that the implementation of Case 2 was experienced more positively when support for the implementation is created through involvement, the improvement initiative is of personal importance, leadership was present and a positive climate was created. The implementation itself was considered successful based the process of the implementation, even though it was not yet implemented into routine care during the exploration of the current study. After completion of the current study, the improvement initiative laid out in Case 2 led to continued work. The results of the effect of the improvement initiative showed a significant improvement in protein intake and an indication of an improvement in hospital length-of-stay (results to be published). Based on these results, we expect that preoperative protein-enriched diet will become part of a bundle of improvement initiatives targeting frail elderly people undergoing surgery. Case 2 showed an improvement initiative targeting preoperative preparation could In contrast, in Case 1 participants felt uninvolved and that their needs were ignored since no room for evaluation was created. Whether the implementation in terms of impact on patient-relevant outcomes was successful could not be determined with this exploration on how improvement initiatives focussing on monitoring value were implemented. The goal of this study was not to reach a certain goal in quantitative terms, but rather explore what went well and what could be improved in the implementation process of VBHC improvement initiatives based on two cases.

**Fig. 3:**
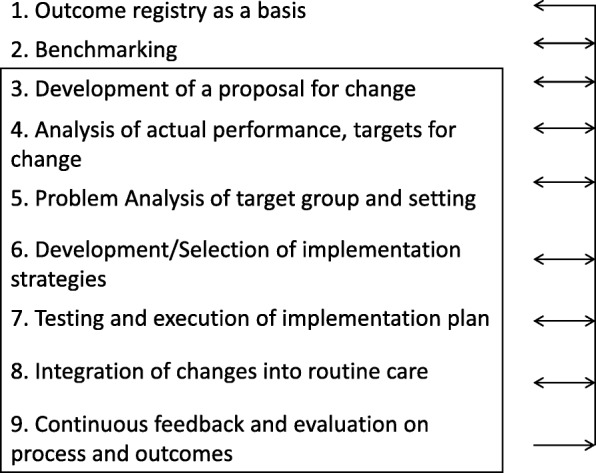
Integrated Implementation Model (IIM) for improvement projects. The steps adapted from the ICM are framed in black. The other steps are new additional steps to the IIM. The arrows on the side indicate the possibility for repetition of steps

Based on the results of this study, we built a framework for the implementation of improvement interventions. From the analysis of the success factors for the implementation of improvement initiatives, it appears that the ICM can add value to VBHC and the implementation of value-based improvement initiatives. We, therefore, propose an implementation model that integrates new steps identified through the interviews that are unique to VBHC in order to add value to the ICM and vice versa (Fig. 3). The Integrated Implementation Model (IIM) consists of two additional steps next to the steps of the ICM. These two steps include: outcome registry as a basis and benchmarking.

Currently, in the ICM there is insufficient focus on the measurement and application of patient-relevant outcomes measures. The final step of the ICM is ‘Continuous feedback and evaluation’ which includes evaluation of performance [14]. Grol et al., however, do not further define performance. Therefore, a focus on outcome measurement is necessary when applying the ICM in the context of VBHC. It is important to consider the proposed implementation model as a roadmap for implementation where at every step of the process possible adaptations need to take place and earlier steps must be repeated. There is support in the literature for our proposal of continuous monitoring of outcomes and adaptation where needed [14].

In the context of VBHC an implementation approach was lacking to guide the implementation of improvement interventions. Whether the IIM adds value to VBHC and vice-versa is yet to be determined and future research should focus on validation of the IIM. However, in the literature, the benefit of new models compared to parallel approached is discussed [43]. The application of an existing, suitable implementation approach is favourable [44]. However, depending on the context and needs a combination of frameworks is necessary [44].

Despite our efforts to rigorously follow the steps of qualitative research, this study has limitations. Firstly, complexity was identified as a limitation. In the first case, doctors needed to change behaviour by adding questions to their usual time-out procedure, which is less complex than targeting the patients as in the second case. Whether an improvement requires doctors or patients to change, can impact support and uptake. As for guideline adherence, guidelines that can be easily understood and are thus less complex have a greater chance of uptake [42]. In our study, strong support and involvement before implementation were identified as important for ensuring successful implementation of complex improvement interventions. Secondly, the first case was part of a larger improvement initiative initiated by another hospital. The initial project included various aspects next to a pre-incision checklist, e.g. the implementation of additional information from actual transoesophageal echocardiography images immediately after induction of anaesthesia [28]. At the current hospital, only a small part of the larger improvement project was implemented, which could have impacted results and motivation for this initiative. Thirdly, comparability of the cases could have influenced the cross-case analysis. The intention was to choose two cases that could be contrasted, yet were also comparable. The cases can be contrasted given the fact that in the first case no structural implementation method was used, whereas in the second case, the ICM was used for the implementation. The cases are comparable, because both initiatives emerged based on outcome measures. However, substantial differences concerning the nature of the cases including the involvement of the health care professionals, the impact on workload, the type of outcome measures and timing, could impact the results. Both cases were implemented as value-based improvement initiatives as an organizational intervention. Fourthly, the composition of the focus group could also potentially impact the results. The focus group consisted of doctors, nurses, data managers and secretaries. Hierarchy could have potentially affected the data [36], as participants may have felt inhibited by the presence of a doctor. Fifthly, the number of interviewed participants was relatively small. Including more participants could have enhanced the description of the cases. However, all possible participants were included in the study. Sixthly, to quantitatively determine the success of the implementation, ideally we would measure the number of safety-checks filled in Case 1 and the number of patients in Case 2 that received protein-enriched diet. However, we did not follow the implementations prospectively, but instead retrospectively analysed the implementation process based on document analysis and interviews. The goal of this study was not to measure success in quantitative terms, but in terms of experience of the implementation process by participants. Seventhly, the data coding was conducted by a single researcher (NZ), which could have posed a threat to the reliability of coding. However, the results of the codes were discussed with three researchers (SG, GW and PvdN) to increase reliability of results. The coding method may have impacted the results of the analysis. Lastly, contamination of both cases may have influenced the implementation process of Case 2 as both interventions were implemented in the same setting. However, both the interventions were implemented at different times (Case 1 between December, 2015 and February 2016 and Case 2 between April 2016 and February, 2017). Since Case 1 preceded Case 2, possible lessons from Case 1 may have influenced the process of Case 2. However, the teams that were involved in both implementations were substantially different.

We aimed to illustrate how a systematic implementation method could support the implementation of improvement interventions based on outcomes. In order to determine the degree of successful implementation, we recommend further studies to evaluate the effect of each case on the health outcomes relevant to the case. We also recommend to test the suggested IIM in a different setting.

## Conclusion

Applying an implementation method such as the ICM which offers guidance for the implementation was found to be valuable for successful implementation. The primary focus for implementation of improvement interventions should be outcome measures because insights into outcomes (that are relevant for patients) give an actual picture of the value added of the improvement initiative. This focus is applicable in general for the ICM, not only in the context of VBHC. We, therefore, propose using the IIM for interventions with the aim of quality improvement. Further research needs to be conducted to evaluate the use of the integrated model.

## Additional file

Additional file 1: Interview guide based on the ICM and VBHC concept. Describes the interview topic guide and questions. (DOCX 15 kb)

## Data Availability

The interview and focus group data analysed are not publicly available due to length and relevance of the interview transcripts but are available from the corresponding author on reasonable request.

## Abbreviations

ICM: Implementation of Change Model
SAVR: Surgical Aortic Valve Replacement
TAVR: Transcatheter Aortic Valve Replacement
TEE: Transesophageal echocardiography
VBHC: Value-based health care
