# Supplementary material for: The implementation of change model adds value to value-based healthcare: a qualitative study
Source: BMC Health Serv Res. 2019 Sep 6;19:643. doi: 10.1186/s12913-019-4498-y (PMC6728951; doi:10.1186/s12913-019-4498-y)
Supplement: Supplementary file 1 — Interview guide based on the ICM and VBHC concept. Describes the interview topic guide and questions. (DOCX 15 kb) [file 12913_2019_4498_MOESM1_ESM.docx]

Additional file 1 Interview guide based on the ICM and VBHC concept

Topic list ISC case

Start questions:

1. What was your role in the implementation?

2. Are there parts of the implementation process that you cannot say anything about due to lack of knowledge about the process?

Elements of the theoretical framework

1. Development of a proposal for change

- Was there a "target" / target for improvement identified at the beginning of the implementation?

- What was the target / goal for improvement?

- How was the target / target identified?

- What was the target based on?

- Was there sufficient support for the goal?

- Was the goal "attractive" enough for a change?

- Was the scope / impact of the goal feasible?

- Has the change proposal met your needs? (personal needs)

2. Analysis of actual performance, targets for change

- Was there a review of the actual performance? This assessment contains questions such as: what kind of care is given? What are the most important deviations from the proposed method?

- Did this analysis lead to a feeling of urgency / interest in the implementation?

- Has this analysis led to a sense of responsibility? Did you yourself feel responsible for the necessary improvement?

- Have concrete targets for improvement been discussed on the basis of this analysis?

- Was this analysis fed back to you?

3. Problem analysis of target group and setting

- Has an analysis been made of the context in which the change should be applied?

- Has an analysis been made about the facilitating or impeding factors for a successful implementation?

- Was the change proposal well communicated?

- Have you felt involved in the implementation of the goal?

4. Development and selection of strategies and measures to change practice / Development or selection of improvement strategies

- Have implementation strategies been developed for successful implementation of the goal / change such as protocols, audit, feedback, computer-aided decision making, patient education, redevelopment of care processes?

- Have strategies been defined for dissemination of the goal / change?

- Was there a protocol / implementation plan shared with you?

- Was a plan for finances worked out? Was this plan shared with you?

5. Development, testing, and execution of an implementation plan

- Was the implementation plan tested in a smaller group?

- Was a pilot carried out?

- Was feedback requested on the implementation plan?

- Was the implementation plan adjusted with feedback?

- Was a proposal made as to how the project could be rolled out further?

- Are you involved in the evaluation of the pilot?

- Was your feedback on the pilot included in adjustments?

6. Integration of changes in routine care

- Have long-term goals been formulated?

- There was clear leadership

- Was there good cooperation?

- Are more people involved in the project if it was needed to spread it further?

7. Continuous evaluation and (possible) adaptions to the plan

- Was feedback regularly requested on the change?

- Has the change / purpose been monitored?

- Was feedback regularly requested on performance?

- Was the project adapted if it was necessary to guarantee sustainability?

- Have short-term, intermediate, and long-term goals been formulated?

Note: Respondents only need to answer questions that apply.
